# Food consumption patterns of adolescents aged 14–16 years in Kolkata, India

**DOI:** 10.1186/s12937-017-0272-3

**Published:** 2017-08-24

**Authors:** Neha Rathi, Lynn Riddell, Anthony Worsley

**Affiliations:** Institute for Physical Activity and Nutrition, Deakin University, 221 Burwood Highway, Burwood, VIC 3125 Australia

**Keywords:** Food habits, India, Adolescents, Gender

## Abstract

**Background:**

The nutrition transition has brought about rapid changes in the structure of the Indian diet. The replacement of traditional home-cooked meals with ready-to-eat, processed foods has contributed to an increased risk of chronic diseases in urban Indians. Improving the nutrition of Indians by promoting healthy food consumption in early life and in adolescence would help to reduce these health risks. However, little is known about the quality and quantity of foods and beverages consumed by urban Indian adolescents. Therefore, the aim of this study was to describe the food consumption patterns in a sample of urban Indian adolescents.

**Methods:**

A self-administered, semi-quantitative, 59-item meal-based food frequency questionnaire (FFQ) was developed to assess the dietary intake of adolescents over the previous day. A total of 1026 students (aged 14–16 years) attending private, English-speaking schools in Kolkata, India completed the survey.

**Results:**

Overall, the adolescents reported poor dietary intakes; over one quarter (30%) reported no consumption of vegetables and 70% reported eating three or more servings of energy-dense snacks, on the previous day. Nearly half of the respondents (45%) did not consume any servings of fruits and 47% reported drinking three or more servings of energy-dense beverages. The mean consumption of food groups in serves/day varied from 0.88 (SD = 1.36) for pulses and legumes to 6.25 (SD = 7.22) for energy-dense snacks. In general, girls had more nutritious dietary intakes than boys.

**Conclusions:**

The Indian adolescents reported poor food consumption patterns, and these findings highlight the need to design effective nutrition promotion strategies to encourage healthy eating in adolescence and targeting food supply and availability.

## Background

Triggered by a complex mix of marketing, social, and economic policies, the Indian nutrition transition [1] has been associated with a significant change in the lifestyles and the dietary habits of urban Indians [2–4]. The rapid proliferation of multinational fast food companies in the Indian food market and the influence of Western culture have replaced traditional home cooked meals with ready-to-eat, processed foods in urban Indian households [5, 6]. These changing food preferences have contributed to the increased risk of chronic degenerative diseases, thus affecting the quality of life and health of about 1.2 billion Indians [2, 6]. This highlights the need to nurture healthy eating habits among Indians from an early age.

Adolescence (10–19 years) is a vulnerable period of life as health-related behaviours that drive the major chronic degenerative diseases start or are reinforced during this time [7]. Adolescents’ food habits are important determinants of both their present and future health [8, 9]. The food intakes of adolescents in developed countries such as USA, UK and Australia do not meet dietary guidelines [10–12]. Adolescents from these countries have high rates of consumption of energy-dense, nutrient-poor foods and inadequate consumption of fruits and vegetables [10–13]. In addition, adolescents also exhibit unhealthy eating habits such as meal skipping and snacking on fast foods [14–16]. Although limited, evidence from developing countries including India also report similar findings [17–20]. These food behaviours may set in train unhealthy eating trends for adult life [8, 9], and contribute to a number of health problems including overweight and obesity, metabolic syndrome, diabetes and a number of cancers [6, 21]. Improvement of the food habits of adolescents is therefore one avenue to reduce the prevalence of these health problems.

Adolescents’ food consumption tends to vary according to gender [22, 23]. Studies across a number of countries have consistently shown that females’ dietary patterns are healthier than those of males [24–26]. Women are more likely to avoid high-fat foods, consume more fruits and fibre and limit salt intake [24] than men. For example in Australia, adolescent girls had a higher average daily intake of fruits compared with male counterparts and boys tended to consume more carbonated beverages than girls [27]. Similarly, British girls preferred eating fruits and vegetables more than boys, whereas boys preferred eating nutrient-poor foods more than girls [28]. Given the consistency of these observations across different cultural groups, it is expected similar dietary patterns may be common among Indian adolescents.

To date, little is known about the food intakes of Indian adolescents. Neither the National Family Health Survey-3 [29] nor the National Sample Survey Office [30] have examined the dietary habits of urban Indian adolescents. This lack of evidence about the food consumption patterns of Indian adolescents is a significant barrier to the development of effective nutrition promotion and disease prevention measures. Therefore, the present study was undertaken to examine the food consumption patterns of a sample of Indian adolescent boys and girls residing in Kolkata city, West Bengal, India.

## Methods

### Research design and sampling

The Dietary and Lifestyle (DAL) survey was conducted among secondary school students attending private schools in Kolkata metropolitan area, India. Convenience sampling informed the selection of nine English-medium (i.e. English speaking) schools (two co-educational schools, two single-sex boys’ schools, and five single-sex girls’ schools). Only year nine students were included in the study because it was expected that these students would have well-developed individual food preferences [31]. Moreover, their academic schedule was less hectic in comparison to those of students in other years, making the administration of the survey more feasible. This cross-sectional survey was reviewed and approved by Deakin University’s Health Ethics Advisory Group (HEAG-H 187_2014). A detailed description of the survey has been reported previously [32].

### Survey instrument

Questions regarding vegetarianism, meal consumption patterns, food and beverage intake, snacking practices, household food rules, home food environment, secondary school nutrition curriculum, school canteen, acquisition of food skills, exposure to media, consumerism, family characteristics and demographics were included in the Dietary and Lifestyle Questionnaire (DALQ). Secondary school students and their nutrition educators in India were extensively involved in the development of this 15-page paper-based questionnaire. Only findings that relate to food and beverage intakes are reported in this paper.

### Development of the FFQ

The FFQ was an integral component of the DALQ. The Childhood Determinants of Adult Health (CDAH) study [33] and the first pass of ASA24-Kids-2014 program [34] informed the structure of the food list i.e. meal categorisation in the FFQ. Both the CDAH study [33] and the ASA24-Kids program [34] use a meal-based enquiry tool which allows respondents to record different meals and snacks consumed at various time intervals during a particular day. A meal-based FFQ accommodates a wide variety of meals and snacks including mixed dishes [35]. Therefore, the current FFQ was designed to provide data on meal patterns across the day and not just overall food intake.

In order to establish the list of meals and snacks included in the FFQ, 54 adolescents (aged 14–15 years) attending a private school in Kolkata were asked to recall the food and beverage items consumed on the previous day for two consecutive days including one weekday and one weekend day. These data were used to inform the development of the meal-based FFQ. As all foods were consumed in frequencies ≥5 occasions by the students, all recalled food items were included in the FFQ (Table 1). The foods reported by the adolescents yielded 59 novel food items for inclusion in the FFQ (Table 1). No food items recorded in the food recalls were omitted from the FFQ food list.

**Table 1:** The list of 59 food and beverage items comprising the FFQ and their reported frequencies

Food items	Frequency ^a^
*Cereals*	
Breakfast cereal	42
Bread/Toast	49
Sandwich	37
Semolina	5
Rice flakes	5
Chapati (Indian bread)	54
Parantha (shallow-fried Indian bread)	24
Luchi/Puri/Kachori (deep-fried Indian bread)	12
Rice	52
Idli (a savoury cake made by steaming a fermented batter of rice and de-husked black lentils)	9
Dosa (a pancake made from a fermented batter of rice and de-husked black lentils)	5
Puffed rice	24
*Pulses and Legumes*	
Pulse dish e.g. lentils, green gram pulse	29
Legume dish e.g. red kidney beans	13
*Vegetables*	
Potato dish e.g. mashed potato	45
Green leafy vegetable dish e.g. spinach curry	19
Other vegetable dish e.g. stuffed bitter gourd, pumpkin curry, vegetable korma (an aromatic and spicy curry with mixed vegetables), vegetable jhalfarezi (a semi-dry recipe with a mix of vegetables cooked in a tomato based gravy).	33
*Fruits*	
Apple	15
Banana	26
Sweet lime	8
Pear	9
Grapes	5
Orange	5
Fruit Juice (Tetrapack/Fresh)	13
*Milk and milk products*	
Milk	49
Lassi (a yogurt-based drink)	13
Cottage cheese (paneer) dish e.g. paneer butter masala (cottage cheese cooked in crème sauce), matar paneer (a preparation of peas and cottage cheese in a tomato based sauce)	28
*Non-vegetarian food products*	
Boiled egg/omelette	20
Egg dish e.g. egg curry,	9
Chicken dish e.g. chilli chicken	12
Fish dish e.g. fish fry	14
Red meat dish e.g. mutton curry	5
*Energy-dense snacks*	
Biscuits/cookies	14
Cake/pastries	23
French fries	39
Pav bhaji (a thick vegetable curry usually prepared in butter and served with a soft bread roll)	25
Samosa (fried triangular-shaped pastry shell with a filling of spiced potatoes, onion, peas, cheese or noodles)	45
Packaged potato chips	40
Pizza	24
Burger	29
Chole bhature (a combination of spicy chick peas and fried breads made of refined wheat flour)	17
Ice cream	24
Ice candy	9
Chocolates	24
Pani puri (fried puff-pastry balls filled with spiced mashed potato, spiced water and tamarind juice)	39
Vegetable roll/wrap	11
Chicken roll/wrap/nuggets	14
Egg roll	5
Momos (a steamed refined wheat flour dumpling filled with meat/vegetables)	19
Indian savoury e.g. bhujia (a crispy deep-fried snack prepared by using gram flour and spices)	12
Popcorn	33
Noodles	39
*Energy-dense beverages*	
Tea e.g. black tea, milk tea, Irish tea	29
Coffee e.g. cold coffee, black coffee	15
Health drink e.g. Bournvita, Horlicks (brand name for health drinks)	44
Soft drink e.g. Sprite, Coke, Pepsi	46
Energy drink e.g. Red Bull, Gatorade	22
Hot chocolate	13
*Water* i.e. Household or school tap water and mineral (bottled) and non-mineral (bottled) water	54

^a^Food frequencies calculated from the food recalls of 54 participants

The 59 food and beverage items were distributed across different time intervals of the day. Seven time slots for the previous day (i.e. 24 h from 6.00 am to 5.59 am) comprising 6.00 am – 9.59 am, 10.00 am – 11.59 am, 12.00 noon – 2.59 pm, 3.00 pm – 5.59 pm, 6.00 pm – 7.59 pm, 8.00 pm – 10.59 pm and 11.00 pm – 5.59 am were included in the FFQ. A number of food items were included twice or three times in the FFQ as they may be consumed across multiple meal or snack occasions. For example, apple was listed three times between 6.00 am and 9.59 am, 10.00 am and 11.59 am, and 6.00 pm and 7.59 pm. Therefore, there was a total of 131 food listings in the FFQ. No identifiers such as ‘Breakfast’ or ‘Lunch’ were assigned to these times.

Three point serving response scales were employed to record the previous day’s food consumption. These response scales were calibrated according to the type of food or beverage. For example, food items like vegetable dishes were assessed in katoris (e.g., ½ katori = 1 serving, 1 katori = 2 servings, and 2 katoris or more = 3 or more servings; katori is Indian equivalent of a bowl). For beverage items like coffee, responses were measured in cups (e.g. ½ cup = 1 serving, 1 cup = 2 servings, and 2 cups or more = 3 or more servings). These serving sizes specified in the FFQ do not relate to any national dietary guidelines.

Pictorial depictions of serving measures i.e. katori (1 katori = 150 ml), glass (1 glass = 250 ml; ½ glass = 1 serving, 1 glass = 2 servings, 2 glasses or more = 3 or more servings) and cup (1 cup = 200 ml) were presented in the questionnaire to aid reporting. Detailed instruction for completing the FFQ was provided: “**Tell us what you ate and drank yesterday.** (Circle foods and beverages consumed by you at different intervals of day from the list given on page No. 2 to page No. 8 (Q6-1 to Q6-131). Also mention the serves (katoris/pcs/glasses/packets) for the food/meal/snacks/drink consumed by you. The serve guide given on the following page will help you in reporting the serves.)” A detailed description of the serving sizes is presented in Table 2.

**Table 2:** Description of serving sizes included in the FFQ

One serving	Two servings	3 or more servings	Food/Beverage items
½ katori	1 katori	2 katoris or more	Breakfast cereal, Semolina, Rice flakes, Rice, Puffed rice, Pulse dish, Legume dish, Pulse dish, Potato dish, Green leafy vegetable dish, Other vegetable dish, Grapes, Cottage cheese dish, French fries, Indian savoury e.g. Bhujia, Popcorn, and Noodles.
1 piece	2 pieces	3 pieces or more	Bread/Toast, Sandwich, Chapati, Parantha, Luchi/Puri/Kachori, Idli, Dosa, Apple, Banana, Sweet lime, Pear, Orange, Boiled egg/omelette, Egg dish, Chicken dish, Fish dish, Red meat dish, Biscuits/cookies, Cakes/pastries, Pav Bhaji, Samosa, Pizza, Burger, Chole bhatura, Chocolates, Pani puri, Vegetable roll/wrap, Chicken roll/wrap/nuggets, Egg roll, and Momos
½ glass	1 glass	2 glasses or more	Fruit juice, Milk, Lassi, Health drink, Soft drink, Hot chocolate, Energy drink, and Water
½ cup	1 cup	2 cups or more	Tea and coffee
½ packet	1 packet	2 packets or more	Packaged potato chips
1 scoop	2 scoops	3 scoops or more	Ice cream
1 stick	2 sticks	3 sticks or more	Ice candy

For the purpose of data analysis, the 59 food and beverage items in the FFQ were classified into nine food groups: cereals, pulses and legumes, vegetables, fruits, milk and milk products, non-vegetarian food products, energy-dense snacks, energy-dense beverages, and water. Out of these nine food groups, six food groups were consistent with food group naming used in Bowman’s FFQ [36]; a validated FFQ suitable for use among urban and rural adult Indian populations [36]. However, this validated FFQ [36] did not include snacks, beverages, and water, therefore, these three food groups were incorporated into the present FFQ.

The food intakes of the adolescents were calculated by summing the serving scores of all the food items within the nine food group categories. For example, for ‘cereals’ consumption, an adolescent who reported consuming two servings (two slices of bread) of bread between 6.00 am and 9.59 am (scored 2), three or more servings of rice between 12.00 noon and 2.59 pm (scored 3), one serving of puffed rice between 3.00 pm and 5.59 pm (scored 1) and one serving of chapatti between 8.00 pm and 10.59 pm (scored 1) was allocated a total score for cereals of 2 + 3 + 1 + 1 = 7 servings/day.

Initially, the FFQ was pre-tested to elicit students’ (*n* = 47; aged 14–16 years) opinions regarding the length, logical structure and readability of the questionnaire to inform further modification of the questionnaire. No changes to the FFQ were made as the participants found the questionnaire readable and positive feedback was obtained.

A total of 37 Year 9 students (15 boys and 22 girls; age: 14–16 years) studying in a private Indian secondary school participated in the test-retest reliability study. The participants completed the FFQ on two occasions, four weeks apart between August and September 2015. The test-retest reliability of the FFQ was assessed using Pearson’s correlation coefficient (r). The test-retest correlations (Pearson’s correlation coefficient) for the food groups ranged from *r* = 0.48 for cereals to *r* = 0.85 for fruits, reflecting fair to moderate reproducibility [37, 38] (Table 3). All correlations were statistically significant (*p* < 0.01).

**Table 3:** Pearson correlation coefficient (r) values for test-retest reliability of food groups included in the FFQ (*n* = 37)

Food group	r (Pearson’s correlation) ^a^
Cereals	0.48
Pulses and Legumes	0.67
Vegetables	0.56
Fruits	0.85
Milk and milk products	0.59
Non-vegetarian food products	0.69
Energy-dense snacks	0.67
Energy-dense beverages	0.62
Water	0.64

^a^All (r) values significant at *p* < 0.01

### Data collection

Prior to the commencement of the DAL survey, the school principals and teachers were given a brief oral introduction to the survey procedures. All year nine students (*n* = 1095) from the nine participating schools were informed about the study through announcements made in the morning assembly. Participation in the survey was voluntary and students were free to opt of the survey anytime. The school authorities distributed the recruitment pack including a Plain Language Statement and a Consent Form to the students. Students (*n* = 1079) with written parental consent were allowed to participate in the survey. The questionnaire was administered to students (*n* = 1026; 53 students were absent on the day of survey) in class time on a weekday (i.e. any day between Tuesday and Friday as per each school’s discretion) under the supervision of class teachers and the lead researcher (NR) between December (winter) 2015 and April (early summer) 2016. The survey was not administered on Mondays because weekly academic tests were scheduled for this day in majority of the participating schools. The students did not receive any gifts for participation.

### Data analysis

The Statistical Package for Social Sciences (SPSS, version 22.0) was used to conduct the data analyses. Descriptive statistics were calculated. Since, the majority of the variables were non-normally distributed, the Mann-Whitney Test, a non-parametric test was used to compare the mean food group scores of the boys and girls. Cross-tabulation and chi-square analyses were performed to investigate the variations in the intakes of individual foods and beverages by gender.

Type 1 error is a common shortcoming associated with population-based surveys [39]. To reduce this error rate, a stringent criterion for determining statistical significance was adopted: an alpha level of *p* < 0.01 was selected to determine statistical significance [39].

## Results

In total, 356 boys and 670 girls completed the survey, the response rate was 95% of eligible students with written parental consent and 94% of all invited students. The socio-demographic characteristics of the respondents are shown in Table 4. The majority of the pupils were within the 14–15 years of age group, the remainder (14%) reported their age as 16 years. Hinduism was followed by 71% of the students.

**Table 4:** Socio-demographic characteristics of students (*n* = 1026)

	Total % (n)	Boys % (n)	Girls % (n)	χ^2^	df	*p*-value
*Gender*	100.0 (1026)	34.7 (356)	65.3 (670)	N.A	N.A.	N.A.
*Age*				36.438	2	<0.001
14 years	44.0 (451)	51.7 (184)	39.9 (267)			
15 years	42.1 (432)	43.0 (153)	41.6 (279)			
16 years	13.9 (143)	5.3 (19)	18.5 (124)			
*Religion*				96.378	8	<0.001
Christianity	0.9 (9)	0.8 (3)	0.9 (6)			
Hinduism	70.7 (725)	77.0 (274)	67.3 (451)			
Islam	16.9 (173)	3.1 (11)	24.2 (162)			
Jainism	7.0 (72)	11.8 (42)	4.5 (30)			
Sikhism	0.8 (8)	1.7 (6)	0.3 (2)			
Zoroastrianism	0.1 (1)	0.0 (0)	0.1 (1)			
Any other	0.3 (3)	0.3 (1)	0.3 (2)			
None	2.1 (22)	2.8 (10)	1.8 (12)			
Don’t know	1.3 (13)	2.5 (9)	0.6 (4)			

*N*.*A* Not Applicable

Among the nine food groups (excluding water), energy-dense snacks was the most commonly consumed food group (6.25 servings/day; SD = 7.22) (Table 5). After this group in decreasing order of average daily serving intake were cereals, energy-dense beverages, vegetables, fruits, milk and milk products, non-vegetarian food products, and pulses and legumes. The adolescents consumed a mean 10.47 (SD = 5.54) servings of water each day. Overall, the Mann-Whitney test results indicate that food intakes of girls significantly varied from boys (Table 5).

**Table 5:** Mean serves/day consumption of food groups (*n* = 1026)

Food group	Total (*n* = 1026) Mean (SD)	Boys (*n* = 356) Mean (SD)	Girls (*n* = 670) Mean (SD)	*p*-value^*^
Cereals	5.52 (3.75)	4.87 (4.30)	5.87 (3.38)	<0.001
Pulses and Legumes	0.88 (1.36)	0.77 (1.32)	0.94 (1.38)	0.014
Vegetables	2.30 (2.25)	1.69 (2.10)	2.62 (2.27)	<0.001
Fruits	2.17 (3.22)	1.35 (2.29)	2.61 (3.54)	<0.001
Milk and milk products	2.07 (2.27)	2.78 (2.54)	1.69 (2.01)	<0.001
Non-vegetarian food products	1.41 (2.14)	1.33 (3.19)	1.46 (1.91)	<0.001
Energy-dense snacks	6.25 (7.22)	7.31 (8.52)	5.68 (6.35)	0.017
Energy-dense beverages	3.96 (5.11)	5.32 (6.53)	3.24 (3.99)	<0.001
Water	10.47 (5.54)	11.69 (5.44)	9.83 (5.49)	<0.001

*p-value obtained with Mann-Whitney Test

Many adolescents failed to consume food items from all eight food groups daily (Table 6). Almost two-thirds (59%) did not consume pulses and legumes. About half (52%) refrained from eating non-vegetarian food products (i.e. egg, fish, chicken, and meat) and a similar proportion did not consume any fruit (45%). Vegetables (30%) and milk and milk products (36%) were not consumed by many students.

**Table 6:** Proportion of adolescents consuming foods from each of the eight food groups (*n* = 1026)^#^

	Total % (n)	Boys % (n)	Girls % (n)	χ^2^	df	*p*-value
*Cereals*				77.209	3	<0.001
No intake	4.8 (49)	10.7 (38)	1.6 (11)			
One serving	2.4 (25)	3.9 (14)	1.6 (11)			
Two servings	10.1 (104)	16.3 (58)	6.9 (46)			
Three or more servings	82.7 (848)	69.1 (246)	89.9 (602)			
*Pulse and Legumes*				7.446	3	0.059
No intake	59.3 (608)	64.9 (231)	56.3 (377)			
One serving	14.1 (145)	11.5 (41)	15.5 (104)			
Two servings	16.4 (168)	14.3 (51)	17.5 (117)			
Three or more servings	10.2 (105)	9.3 (33)	10.7 (72)			
*Vegetables*				71.340	3	<0.001
No intake	30.1 (309)	44.1 (157)	22.7 (152)			
One serving	11.9 (122)	8.4 (30)	13.7 (92)			
Two servings	17.5 (180)	21.1 (75)	15.7 (105)			
Three or more servings	40.4 (415)	26.4 (94)	47.9 (321)			
*Fruits*				54.134	3	<0.001
No intake	45.3 (465)	56.2 (200)	39.6 (265)			
One serving	7.9 (81)	11.2 (40)	6.1 (41)			
Two servings	15.9 (163)	15.2 (54)	16.3 (109)			
Three or more servings	30.9 (317)	17.4 (62)	38.1 (255)			
*Milk and milk products*				48.440	3	<0.001
No intake	36.4 (373)	26.4 (94)	41.6 (279)			
One serving	7.0 (72)	4.8 (17)	8.2 (55)			
Two servings	26.2 (269)	25.6 (91)	26.6 (178)			
Three or more servings	30.4 (312)	43.3 (154)	23.6 (158)			
*Non-vegetarian food products*				35.773	3	<0.001
No intake	51.9 (533)	64.0 (228)	45.5 (305)			
One serving	12.8 (131)	7.6 (27)	15.5 (104)			
Two servings	13.5 (138)	9.3 (33)	15.7 (105)			
Three or more servings	21.8 (224)	19.1 (68)	23.3 (156)			
*Energy-dense snacks*				0.543	3	0.909
No intake	9.7 (100)	9.6 (34)	9.9 (66)			
One serving	7.7 (79)	7.0 (25)	8.1 (54)			
Two servings	12.0 (123)	12.6 (45)	11.6 (78)			
Three or more servings	70.6 (724)	70.8 (252)	70.4 (472)			
*Energy-dense beverages*				21.709	3	<0.001
No intake	22.7 (233)	17.7 (63)	25.4 (170)			
One serving	5.8 (59)	4.2 (15)	6.6 (44)			
Two servings	24.9 (255)	21.6 (77)	26.6 (178)			
Three or more servings	46.7 (479)	56.5 (201)	41.5 (278)			

^#^Examples of servings given below

A. For food items likes like pulses and legumes, servings were assessed katoris (e.g., ½ katori = 1 serving, 1 katori = 2 servings, and 2 katoris or more = 3 or more servings; katori is the Indian equivalent of a bowl)

B. For beverage items like tea, servings were assessed in cups (e.g. ½ cup = 1 serving, 1 cup = 2 servings, and 2 cups or more = 3 or more servings)

There were gender differences in the numbers of servings consumed from the eight different food groups. More females consumed cereals, vegetables, fruits and non-vegetarian food products than their male counterparts (Table 6, *p* < 0.001). In contrast, more males consumed three or more servings of milk and milk products and energy-dense beverages (Table 6, *p* <0.001). However, there were no significant gender differences in the consumption of pulses and legumes (*p* = 0.059) or energy-dense snacks (*p* =0.909).

## Discussion

The present findings highlight the overconsumption of energy-dense, nutrient-poor foods and under consumption of vegetables, pulses and animal foods among this sample of adolescents residing in Kolkata City, India. These unhealthy dietary intakes may increase the risk of nutrient inadequacy [40] and weight gain among nutritionally vulnerable Indian adolescents [41–44]. Considering the likely tracking of food behaviours into adulthood [8, 45], there is an immediate need to modify such behaviours during this pubertal phase, thus, enabling adolescents to develop healthy food practices for their adulthood.

As a consequence of food globalisation, the consumption of energy-dense and nutrient-poor foods and sugar-sweetened beverages has increased substantially, particularly, in urban regions [6]. This overconsumption is quite evident in the present study as nearly three quarters of the sample consumed three or more servings of energy-dense snacks and about half of the respondents consumed three or more servings of energy-dense beverages. Singh and colleagues also found that about one third (32.1%) of secondary school students (*n* = 510; aged 12–18 years) in New Delhi area consumed fast food (e.g. burgers, pizzas, fried foods etc.) three or more times per week [46].

Because of their hyper palatability, attractiveness and ready-to-eat attributes [47], these non-essential foods are becoming a frequent and dominant component of adolescents’ diets in most economically developed countries [22, 48–50] as well as developing countries like India [51, 52]. Data from the current study further support these observations. Frequent consumption of non-essential foods may contribute to a variety of negative health outcomes [53], including obesity [54], insulin resistance [55] and heart disease [56]. To prevent these diet-related chronic diseases, energy-dense snacks and beverages should be consumed sparingly or not all [6].

The study participants consumed a mean of 2.3 serves/day of vegetables. This is similar to findings from an Australian study which found that adolescents (aged 14–16 years) consumed 2.0 and 2.2 serves/day of vegetables between 1995 and 2007 [57]. Gupta and colleagues reported that secondary school girls (aged 13–15 years) of New Delhi, India consumed 175 g/day of vegetables (both green leafy and other vegetables) [58]. The Indian dietary guidelines recommend a daily intake of three portions of vegetables (1 portion of green leafy vegetable and 2 portions of other vegetables; 1 portion = 100 g) for adolescents (aged 10–18 years) [6]. In light of this recommendation, it appears that the study participants had vegetable intakes below current national guidelines.

Almost half of the students (45%) reported consuming no serves of fruit over the previous day. Again, this is consistent with other evidence. For example, the Global School-Based Health Survey data from 16,084 adolescents (aged: 13–15 years) from five Southeast Asian countries (India, Indonesia, Myanmar, Sri Lanka and Thailand) suggest that nearly a quarter of the sample (28%) reported consuming fruits less than once per day [59]. Likewise, Deka and colleagues found that 52% of the adolescents (*n* = 400; aged: 10–19 tears) residing in Jhansi district, Uttar Pradesh, India did not consume any fruit on daily basis [17]. Both the present and previous findings underscore the need for encouraging daily fruit consumption among adolescents.

Unfortunately the students also reported low intakes of pulses and legumes (0.88 serves/day) and cereals (5.52 serves/day). Nationwide surveys also echo similar findings [60, 61]. Such poor intakes could be attributed to the progressive decrease in per capita availability of pulses from 69 g 1961 to 32 g in 2005 [62]. Typically, cereals form the staple diet of the Indian population [3]; however, cereal consumption by Indians has waned in recent decades, with a substantial proportion of the population failing to meet cereal intake recommendations [3, 63]. At the national level, cereal intake dropped from 353 g to 331 g/person/day in the urban areas and from 447 g to 404 g/person/day in rural areas over the period of 1993–94 to 2004–05 [60]. Similar decline in the consumption of whole grains have also been reported in French children, adolescents and adults [64]. This declining trend in cereal consumption has been accompanied by a shift towards energy-dense foods as a source of energy in diets [3, 6]. Perhaps, these inadequate intakes could be a reflection of the global decline in per capita cereal output from 335 kg per year in 1980–1985 to 310 kg by 2000–2005 [62].

India is the largest producer of milk in the world [62]; however, nearly two-fifths (36%) of the participants did not report any consumption of milk and milk products. Comparatively, only a small proportion (4.6%) of Indian children and adolescents (*n* = 1000; aged: 5–18 years) living in Jaipur, Rajasthan failed to consume milk and milk products on a daily basis [19]. In line with this, one-tenth of the Jordanian adolescents (*n* = 302; aged: 11–18 years) also reported no serving of milk consumption [65]. Nevertheless, the consumption of milk and dairy products among adolescents has diminished considerably in industrialised nations over the recent past [66].

Over half of the respondents (52%) did not consume meat and meat derivatives. Similar proportion (51%) of rural adolescents (*n* = 150; aged: 11–19 years) from Himachal Pradesh, India followed a vegetarian diet i.e. they did not consume egg, fish, and meat [67]. The present sample was dominated by Hindu population (71%) and this could partly explain the low intakes of fish, meat, and poultry. This dominance of vegetarianism in the Indian culture [68] is triggered by religious beliefs i.e. Hinduism, Jainism and Buddhism supports abstinence from meat and meat derivatives [69]. A recent literature review describing trends in food and nutrition intake patterns in the different states of India indicates that majority of the Indians are vegetarians and animal foods are generally consumed less frequently [68]. One the other hand, evidence from predominantly meat-eating countries like Bangladesh [70], Pakistan [71], Bahrain [72], USA [73] and northern European nations [13] show high popularity of meat and meat products among adolescents.

Indian vegetarians mostly derive their protein from milk and its products as well as pulses and legumes [3]. Nonetheless, low intakes of pulses and legumes as well as other protein rich food sources may increase the underlying disease burden from undernutrition and micronutrient deficiency [74–78]. Considering the enduring prevalence of nutritional deficiencies, Indian adolescents should be encouraged to consume foods rich in micronutrients like pulses, fruits, vegetables, oilseeds and animal foods [68, 79].

More girls in the present study demonstrated food habits that were more closely aligned with the national and international dietary guidelines [80, 6, 81] than the boys. Previous studies reflect similar gender differences observed in economically developed countries [22, 28, 82]. These gender differences could be attributed to greater health consciousness among women [24]. Moreover, compared to male adolescents, adolescent women may be more concerned with weight-control behaviours [83]. In addition, the masculinity literature suggests that masculine ideologies and norms play a significant role in discouraging men from eating healthily [84–86].

The present study provides new evidence about the food intake patterns of adolescents in Kolkata City. An important message from the above findings is that dietary intakes in these adolescents consist of excess intakes of energy-dense, nutrient-poor foods and inadequate intakes of nutrient-dense foods. This supports the need for healthy eating initiatives aimed at increasing the consumption of fruits and vegetables, milk products, pulses and legumes while decreasing the consumption of energy-dense, nutrient-poor foods, and sugar-sweetened beverages in adolescents.

The health benefits associated with the consumption of nutritious foods like fruits, vegetables, and dairy products have been published widely [87–90]. For example, vegetable intake is linked to reduced cardiovascular, cancer and all-cause mortality [88]. Similarly, regular fruit consumption reduces the risk of developing chronic diseases [89, 91]. Milk and milk products provide energy, protein, micronutrients and bioactive compounds essential for bone and dental integrity and maintenance of healthy body composition [66]. A systematic review and meta-analysis of dairy intake and adiposity suggest a modestly protective effect on adiposity during adolescence [90]. These numerous positive health outcomes highlight the significance of incorporating nutritious foods in adolescents’ daily diets.

Therefore, in order to encourage healthy eating among adolescents, Indian schools could perhaps incorporate the newly evolved food literacy concept [92] into its academic curriculum as food literacy has the potential of increasing fruit and vegetable intake in teenagers [93, 94]. An additional way to improve the dietary habits of adolescents may be through the implementation of effective school canteen policies. Healthy school canteen policies have been successful in improving the availability, accessibility, variety and affordability of healthy food choices in canteens [21, 95–97], thus supporting students in consuming nutritious foods.

### Strengths and limitations

The present findings need to be treated with caution since the self-administered FFQ was limited to food and drink items predominantly consumed in West Bengal and other parts of eastern India and do not represent all the foods eaten in other parts of India. Direct comparisons could not be drawn between the present findings and previous findings as different dietary assessment methodologies were employed. Moreover, the food items comprising the food groups in the current survey may not be identical to those used in other dietary surveys. Hitherto, studies have commonly used FFQ to monitor dietary assessment over a period of time (e.g. month) [22, 36] or 24 h dietary recall for estimating previous day’s food intake [98, 99]. However, the present study employed a novel dietary assessment technique - a meal-based FFQ was developed to assess the frequency of servings of food and beverage items consumed over the last 24 h. The use of convenience sampling might have affected the generalisability of the present findings as the study sample may not be representative of all the private secondary school students in the Kolkata metropolitan area, public school students or adolescents in other parts of India. However, considering, the high prevalence of overweight and obesity among private school adolescents [41], this cross-sectional survey was carried out in private schools. Moreover, private schools are responsible for providing education to 40% adolescents in urban India [100].

In addition, the present findings may be confounded by the seasonal variation in dietary intakes and social desirability bias. The use of self-administered FFQ could also pose certain limitations as dietary recall is dependent on the memory, literacy and numerical skills of the respondent [35, 101]. The FFQ also did not classify cereals as whole grains or refined cereals. Because of logistic limitations, criterion or convergent validity [35, 101] of the FFQ was not examined in this study. However, the FFQ was developed through extensive collaboration with students and therefore has high face validity, adding strength to the study. Moreover, the observed sex differences in reported food intake reflect known sex differences in food intakes [22, 28, 82] supporting the discriminative validity of the FFQ. Nevertheless, further validation of this FFQ is required. Another limitation is lack of data on demographic characteristics like weight status, body mass index (BMI), socio-economic status (SES) of parents and country of birth. Future research could explore the impact of these characteristics on Indian adolescents’ food consumption patterns. Regardless of these shortcomings, the high response rate, the large sample size, and the uniqueness of the dietary assessment tool, tailored to adolescents’ food habits, form the strengths of the study [22, 28, 82].

## Conclusions

Overall, adolescents attending private secondary schools in Kolkata demonstrate unhealthy dietary patterns. Their frequent consumption of energy-dense, nutrient-poor foods and sugar-sweetened beverages and the omission of a variety of healthy foods from their daily diets puts them at risk of developing chronic degenerative diseases. In the light of the increasing prevalence of obesity and diet-related diseases, actions should be taken to enable adolescents to eat more healthily. These include effective public health initiatives such as the adoption of healthy school food policies and food literacy curricula to foster healthy eating habits among India’s 238 million adolescents.

## Abbreviations

BMI: Body mass index
DAL: Dietary and Lifestyle
DALQ: Dietary and Lifestyle Questionnaire
FFQ: Food frequency questionnaire
HEAG: Health Ethics Advisory Group
SES: Socio-economic status
